# Graphene-Based Nanomaterials as Efficient Peroxidase Mimetic Catalysts for Biosensing Applications: An Overview

**DOI:** 10.3390/molecules200814155

**Published:** 2015-08-04

**Authors:** Bhaskar Garg, Tanuja Bisht, Yong-Chien Ling

**Affiliations:** 1 Department of Chemistry, National Tsing Hua University, Hsinchu 30013, Taiwan; 2 Department of Chemistry, Government Degree College, Champawat 262523, Uttarakhand, India; E-Mail: tanuja.bisht16@gmail.com

**Keywords:** graphene, graphene oxide, graphene-based nanomaterials, peroxidases, enzyme mimetics, heterogeneous catalysts, biosensing and diagnostics, tetramethylbenzidine, hydrogen peroxide

## Abstract

“Artificial enzymes”, a term coined by Breslow for enzyme mimics is an exciting and promising branch of biomimetic chemistry aiming to imitate the general and essential principles of natural enzymes using a variety of alternative materials including heterogeneous catalysts. Peroxidase enzymes represent a large family of oxidoreductases that typically catalyze biological reactions with high substrate affinity and specificity under relatively mild conditions and thus offer a wide range of practical applications in many areas of science. The increasing understanding of general principles as well as intrinsic drawbacks such as low operational stability, high cost, difficulty in purification and storage, and sensitivity of catalytic activity towards atmospheric conditions of peroxidases has triggered a dynamic field in nanotechnology, biochemical, and material science that aims at joining the better of three worlds by combining the concept adapted from nature with the processability of catalytically active graphene-based nanomaterials (G-NMs) as excellent peroxidase mimetic catalysts. This comprehensive review discusses an up-to-date synthesis, kinetics, mechanisms, and biosensing applications of a variety of G-NMs that have been explored as promising catalysts to mimic natural peroxidases.

## 1. Introduction

Nature is the greatest engineer and the main source of all inspirations throughout. Biologically inspired designs or an adaptation from nature and an attempt to incorporate such ideas into better solutions for technological issues and sustainable living is commonly known as “biomimetics” or, “biomimicry”, “bionics”, and “biognosis”. In general, biomimetics simply refers “*mimicking the nature or biology*”. The term “biomimetics” is derived from the Greek word biomimesis and was coined by Otto Schmitt [1] whose doctoral work was focused on the development of a physical device that could unequivocally mimic the electrical action of a nerve. However, the word “biomimetics” made its first public appearance in 1974 in Webster’s dictionary and is defined as “the study of the formation, structure, or function of biologically produced substances and materials (as enzymes or silk) and biological mechanisms and processes (as protein synthesis or photosynthesis) especially for the purpose of synthesizing similar products by artificial mechanisms which mimic natural ones”. Thus the research field of biomimetics is highly multidisciplinary, drawing from many areas of science, including, but not limited to, (bio)chemistry, molecular biology, catalysis, nanotechnology, material science, engineering, physiology, immunology, biosensing, food technology, and environmental science.

Natural enzymes, most of which are proteins, represent linear chains of amino acids that can fold and self-assemble, by making the use of multivalent interactions to produce a three-dimensional structure, a factor responsible for enzymes’ specificity. Natural enzymes, adjudicating many biological processes in living organisms, are exquisite biocatalysts that exhibit very high catalytic activity (up to 10^19^ times for a specific substrate or reaction) with high substrate specificity and selectivity under relatively mild conditions (pH, temperature, and pressure) [2,3]. Despite this, on exposure to chemical denaturants, inhibitors, or relatively harsh environmental conditions, the enzyme structures unfold (denature) leading instability to the structure, which in turn results in a loss of their catalytic activity. In addition, the high cost in preparation, purification, and storage of natural enzymes also restricts their wide technological applications. Consequently, the pursuit of alternative molecules or materials that can achieve most of the above-mentioned challenges has resulted in the introduction of man-made enzymes or artificial enzymes [4,5,6,7,8,9,10].

“Artificial enzymes”, a term coined by Ronald Breslow [5] for enzyme mimics, is an exciting and promising branch of biomimetics, which relies on the use of highly efficient alternative materials including supramolecular catalysts following the general and essential principles of natural enzymes. A conventional route to discover an artificial enzyme is to reproduce the intangible structure of enzyme active sites, the model enzymes. Alternatively, the functions of the enzymes can be imitated without copying its structure (enzyme mimics). In the late 1950s, the non-covalent interactions were the driving forces to produce artificial systems containing enzymes-like properties [11,12]. In the same decade, Sumner was awarded Nobel Prize for his discovery that enzymes can be crystallized [13]. In 1965, cyclodextrin inclusion compounds were evaluated as the first hosts to imitate enzyme hydrophobic pocket at small molecular level [4,14]. However, it was pioneering work by Breslow and Tabushi [5,15,16,17] which ignited considerable research effort, focusing on the development of a range of structurally diverse biomolecules to mimic the structures and functions of natural enzymes [18,19,20,21,22,23]. It would be worthwhile to mention here that the use of the lock-and-key principle of enzymes was the main source of inspiration in featuring the early examples of supramolecular catalysis [5,15,16,17].

Over the last decade, the research interests in enzyme mimics have expanded from molecular to the nanoscale resulting in the formation of highly organized biological materials. The organization of nanomaterials (NMs) in a hierarchical manner with intricate nanoarchitecture provides a significant impetus in mimicking nature using nanofabrication techniques. In general, a complex interplay between the surface structures, morphology, and physicochemical properties results in the formation of multi-functional materials having outstanding properties. Though imitating natural enzymes with NMs appears unreasonable because they are as different as balls and strikes (most natural enzymes are proteins having a well-defined tertiary structures, whereas, due to their different sizes and shapes, most NMs are not uniform anatomically; furthermore, natural enzymes are not as hard and crystalline as NMs), yet they do share certain similarities in overall surface charge, size, and shape. Besides, the external surface of NMs can be functionalized with desirable functional groups (similar to those expressed by natural enzymes), enabling them the materials-of-choice for potential enzyme mimics.

NMs are small-sized chemical entities with large surface area, exhibiting characteristic physicochemical and biological properties that are significantly different from those of the same material in bulk form. Historically, for instance, gold has been considered as highly inert material with little or no reactivity. On the other hand, gold nanoparticles (AuNPs) have been evaluated as highly efficient catalysts for a variety of chemical transformations under different reaction conditions. In the pioneering work, Scrimin, Pasquato, and co-workers [24] discovered that surface functionalized nano gold clusters can function as powerful catalyst for the transphosphorylation of 2-hydroxypropyl-4-nitrophenyl phosphate (HPNPP), a RNA-model substrate. Owing to their outstanding catalytic properties, such gold nanostructures were christened as “nanozyme” by Scrimin, Pasquato, and co-workers in analogy to the “synzyme”, nomenclature used for polymers with enzyme-like activity [6].

Indubitably, due to the marriage of nanotechnology with biology, the last decade has witnessed substantial advancement in designing a variety of functional nanoscale materials as highly active enzyme-mimetic catalysts. In this context, literature reports highlighting the use of, but not limited to, AuNPs [25], cerium oxide NPs [26,27,28], magnetic NPs [8,29], platinum NMs [30,31], manganese dioxide NMs [32], bimetallic nanostructures [33], and carbon-based NMs [34,35,36,37,38,39] are particularly notable. Owing to their unique surface characteristics, the above-mentioned NMs have been proven as excellent biomimetic catalysts for a variety of enzyme mimics, namely, peroxidase, oxidase, catalase, and superoxide dismutase, demonstrating their wide applications in biosensing, environmental protection, immunoassays, and theranostics.

Effective as these nanostructures are, there is no doubt that 21st century is the era of graphene, one of the most studied NMs, and the scope of its utility has grown remarkably over the past decade. The contemporary applications of graphene now intersect a variety of disciplines including biomimetics and heterogeneous catalysis [40,41,42,43,44,45,46]. Indeed, the fabrication of graphene or its subtypes such as graphene oxide (GO) and reduced graphene oxide (rGO) with a range of nanoscale structures have opened new opportunities in developing highly efficient functional NMs with catalytic performances similar to or even better than that of natural enzymes, in particular, peroxidases. Although the activity of carbon-based NMs as nanozymes have been partially covered in some early reviews [39,47,48] yet to the best of our knowledge, graphene-based NMs (G-NMs) as peroxidase mimetic catalysts have not been reviewed by any research group so far and it is, therefore, worthwhile to shed light on this fast-growing field. As such, this review highlights the years’ efforts in designing, fabricating, and exploring GO, graphene, and a range of G-NMs as peroxidase mimetics with remarkable performances. The scope intends to cover, not only the synthesis, kinetics, and mechanisms, but also their potential applications in biosensing and other relevant areas. Finally, both potential issues and future perspectives of G-NMs are discussed for further growth and study in the field.

## 2. An Overview of Peroxidases

Peroxidases (EC 1.11.1.7) represent a large family of isoenzymes found in almost all living organisms. In general, peroxidases are heme proteins containing iron(III) protoporphyrin IX or ferriprotoporphyrin IX (four pyrrole rings are coordinated to Fe(III)) as the prosthetic group. Peroxidases may be categorized as mammalian and plant peroxidases ranging in molecular weight from 35,000 to 100,000 Da [49]. Nevertheless, mammalian peroxidases are relatively much larger than their plant analogues and can have *ca.* 576–738 amino acids. In mammals, peroxidases play a key role in different metabolic activities and thus their levels in human body can directly or indirectly act as a biomarker in a variety of diseases.

**Scheme 1:**
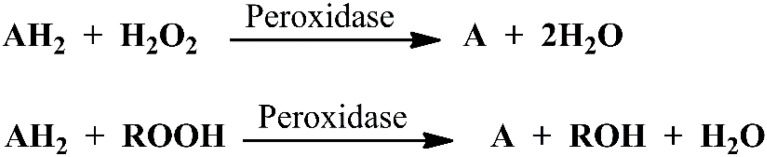
Representative reactions catalyzed by peroxidase.

Peroxidases or a group of oxidoreductases typically catalyze biological reactions, in which peroxides such as hydrogen peroxide (H_2_O_2_) and alkyl hydroperoxide (ROOH) are reduced, while a redox substrate acting as an electron donor is oxidized (Scheme 1). It should be noted that the nature of the electron donor is very much dependent on the structure of the enzyme. Through this catalysis, peroxidases can scavenge H_2_O_2_, a naturally occurring by product of oxygen metabolism in human body, resulting in the formation of water and oxygen. In this way, peroxidases play an important role as highly efficient antioxidant defense systems to combat complications engendered by reactive oxygen species [50].

Nicotinamide adenine dinucleotide (NADH) peroxidase (EC 1.11.1.1), nicotinamide adenine dinucleotide (NADPH) phosphate peroxidase (EC 1.11.1.2), cytochrome-*c* peroxidase (EC 1.11.1.5), horseradish peroxidase (HRP) (EC 1.11.1.7), iodide peroxidase (EC 1.11.1.8), and glutathione peroxidases (GPx) (EC 1.11.1.9) constitute the familiar examples among all peroxidases. The latter have been known, chiefly, to be selenium-containing enzymes encompasses a family of eight isoenzymes (GPx1-8) having varied distribution in the human body with diverse functions. However, GPx1 is the most abundant isoenzyme found in the cytoplasm of nearly all mammalian tissue and protect the organism from oxidative damage [51].

Aside from those of GPx, HRP is the subject of exceptional interest among scientific community and has been studied for more than a century [52]. This interest is reflected in the published work, comprising thousands of research papers on HRP in the literature (~49,678 publications @SciFinder^®^ 2015; key word: horseradish peroxidase). Needless to say, as the list continues to expand, one can be easily overwhelmed by the collection of this vast knowledge. Although the term HRP is somewhat used generically, the roots of horseradish (*Armoracia rusticana*), a hardy perennial herb cultivated in temperate regions, contain a range of characteristic peroxidase isoenzymes, of which the C isoenzyme, HRP C, is the most abundant and routinely used in clinical and bioanalytical chemistry as well as a reagent in organic synthesis, covering hydroxylation, oxidative coupling, oxygen transfer, and *N*- and *O*-dealkylation reactions [53]. Because of the oxidative nature of peroxidases, they offer a wide range of practical applications in many areas as summarized in Table 1 with selected citations.

**Table 1:** The applications of peroxidases ^a^.

Types of Peroxidases	Application(s)	References
LiP, MnP, and HRP	Dye decolorization	[54,55,56]
HRP, LiP, MnP, and microbial peroxidase	Bioremediation of waste water: removal of phenolic and amine contaminants	[57,58,59,60]
HRP	Deodorization of swine slurry	[61]
Fungal peroxidases	Degradation of lignocellulosic biomass: biofuel production	[62]
HRP	Detection of antigens or antibodies: ELISA	[63,64]
LiP	Biopulping: delignification of wood pulp	[65]
Fungal peroxidases	Transformation of pesticides	[66,67]
Chloroperoxidase		
LiP, MnP	Bioremediation of polycyclic aromatic hydrocarbons	[68,69]
HRP	Biosensing and diagnostics: catalysis	[64,70,71]
Plant peroxidases		
HRP	Organic and polymer synthesis	[52,72]
HRP, GPx, TPO, LPO, SPO, MPO, EPO, and uterine peroxidase	Cancer therapy and pathological applications	[52,73]

^a^ LiP, Lignin peroxidase; MnP, Manganese peroxidase; HRP, Horseradish peroxidase; GPx, Glutathione peroxidases; TPO, Thyroid peroxidase; LPO, Lactoperoxidase; SPO, Salivary peroxidase; MPO, Myeloperoxidase; EPO, Eosinophil peroxidase, and ELISA, Enzyme-linked immunosorbent assays.

**Scheme 2:**
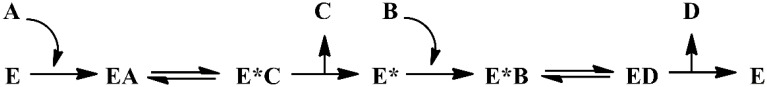
A schematic of ping-pong mechanism or double displacement reaction of peroxidases.

In general, the peroxidase activity, for instance, HRP is the result of a non-sequential mechanism (ping-pong). The ping-pong mechanism, also known as double-displacement reaction, is characterized by the change of the enzyme (bounces back and forth) from an intermediate state to its standard state just like a ping-pong ball. Another key feature of the ping-pong mechanism is that one substrate is converted to the product and dissociates or releases before the second substrate binds. Scheme 2 explains the ping-pong mechanism via an enzymatic action, where A and B are two substrates, C and D are products, and E and E* denote enzyme in standard and intermediate states, respectively.

**Figure 1:**
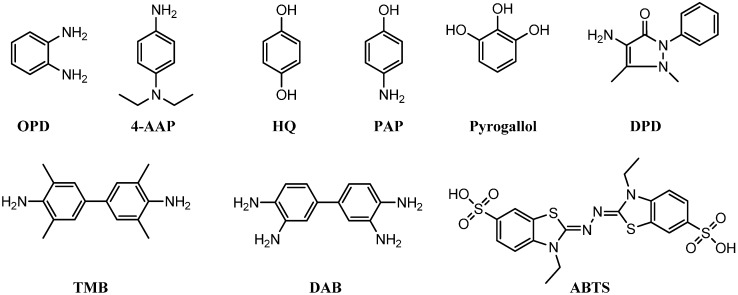
Chemical structures of chromophore substrates commonly used to study the peroxidation reactions.

To detect peroxidase-like activity of nanoscale structures in a specific reaction, both the formation of oxidized product and consumption of H_2_O_2_ are evaluated. The concentration of the latter can be directly measured through a color change of certain chromophores. For instance, in the presence of H_2_O_2_, the oxidation of 2,2′-azino-*bis*-(3-ethylbenzothiozoline-6-sulfonic acid) (ABTS), 3,3′,5,5′-tetramethylbenzidine (TMB), and *o*-phenylenediamine (OPD), in general, produces a green, blue, and yellow color, respectively. Aside from those, 4-aminoatipyrene (4-AAP)-phenol, hydroquinone (HQ), amplex red (AR), *p*-aminophenol (PAP), 1,2,3-trihydroxybenzene (THB) or pyrogallol, 3,3′-diaminobenzidine (DAB) and *N*,*N*-diethyl-*p*-phenylenediamine sulfate (DPD) constitute the additional examples of potential chromophores being used in peroxidase mimics (Figure 1).

Generally, the rate of the reaction is determined by monitoring the absorbance intensity changes as a function of time. Nevertheless, if the substrate oxidation is associated with some fluorescent markers such as rhodamine B and/or quantum dots (QDs), fluorescence emission spectroscopy can be used. At a fixed concentration of catalyst, several kinetic runs are usually performed with varying concentration of chromophores or H_2_O_2_ and vice versa. The kinetic parameters such as maximum turnover number (*K*_cat_), reaction velocity (ν_max_), and Michaelis–Menten constant (*K*_m_) are then determined by straight line plots of variations of the Michaelis–Menten equation, such as Lineweaver–Burk Plot [74].

In an elegant work, Yan and co-workers [8] discovered that ferromagnetic metal NPs (Fe_3_O_4_) with different sizes of 30, 150, and 300 nm possess intrinsic peroxidase-like activity. Specifically, under the optimized conditions (4–90 °C, pH = 0–12), all sized MNPs catalyzed the reactions of TMB, OPD, and diazo aminobenzene (DAB) substrates in the presence of H_2_O_2_, producing blue, orange, and brown colors, respectively. Interestingly, with TMB substrate, Fe_3_O_4_ NPs exhibited relatively higher catalytic activity than HRP. Nevertheless, the catalytic activity of Fe_3_O_4_ NPs was also found to be dependent on H_2_O_2_ concentration, pH, and temperature, similar to that of HRP. Based on these findings, a novel immunoassay, providing three-in-one functions, was developed effectively. This study set the new trend and opened the door for the development of diversified nanoscale materials as peroxidase mimetics for a wide range of potential applications.

## 3. Peroxidase Mimic@ Graphene-Based Nanomaterials (G-NMs)

### 3.1. Graphene Oxide, Graphene, and/or Reduced Graphene Oxide as Peroxidase Mimetic Catalysts

GO, an oxidation product from graphite, largely exists as mono-, bi- or at most a few layers of graphene sheets consisting of a complex cocktail of oxygen-rich functional groups (-COOH, C-O-C, and -OH). To appreciate the role that GO can play in peroxidase mimics, it is worthwhile to shed light on the catalytic performances of other classical and non-classical carboxyl-functionalized nanocarbon oxides, exhibiting peroxidase-like activity. Table 2 summarizes the peroxidase-like activity of a variety of nanocarbon oxides, from fullerenes to carbon nanotubes and mesoporous carbon-to-carbon nanodots. It must be pointed out that the peroxidase-like activity of most nanocarbon oxides has been connected to their intrinsic properties, for instance, the presence of carboxyl groups in the aromatic domains [75] rather than any metal impurity.

**Table 2:** Peroxidase-like activity of nanocarbon oxides ^a^.

Nanocarbon Oxides	Method	Substrate	LOD	Applications	Ref.
Fullerene oxide {C_60_[C(COOH)_2_]_2_}	Colorimetric	TMB	0.5 μM	Glucose detection	[76]
SWCNTs oxide	Colorimetric	TMB	1 nM	SNP detection	[36]
CFMP	Colorimetric	TMB	0.4 μM	H_2_O_2_ detection	[77]
CNDs	Colorimetric	TMB, OPD, and pyrogallol	0.2 μM	H_2_O_2_, glucose detection	[78]
			0.4 μM		
CDs	Colorimetric	MO and MR	–	Degradation of dyes	[79]

^a^ LOD, Limit of detection; TMB, 3,3′,5,5′-tetramethylbenzidine; SWCNTs, Single-walled carbon nanotubes; SNP, Single nucleotide polymorphism; CFMP, Carboxyl functionalized mesoporous polymer; CNDs, Carbon nanodots; CDs, Carbon nanodots as per ref. [79]; MO, Methyl orange; MR, Methyl red.

Present day, the peroxidase mimetic chemistry of G-NMs is largely inherited from Qu’s pioneering work on SWCNTs [36]. In their follow-up work, the group discovered that single-layered GO, free from metallic impurities, possess intrinsic peroxidase-like activity and its catalysis is strongly dependent on pH, temperature, and H_2_O_2_ concentration, similar to HRP [37]. Specifically, the reaction of TMB and H_2_O_2_ in the presence of GO produced a blue color solution of 3,3′,5,5′-tetramethylbenzidine diimine (TMBDI), a diagnostic for peroxidase-like catalytic activity. Due to its very high surface-to-volume ratio and high affinity to TMB (*K*_m_ 0.0237 mM) than HRP (*K*_m_ 0.275 mM), GO exhibited higher catalytic activity and the catalytic reaction followed a typical ping-pong mechanism (parallel lines of double-reciprocal plots). Further, it was proposed that due to the interaction between GO, TMB, and H_2_O_2_, a charge-transfer n-type doping of graphene domain increases the Fermi level and thus the electrochemical potential from the lowest unoccupied molecular orbital (LUMO) of H_2_O_2_ accelerates the electron transfer from graphene domain to H_2_O_2_, providing a high density of catalytically active centers for binding redox substrate.

**Scheme 3:**
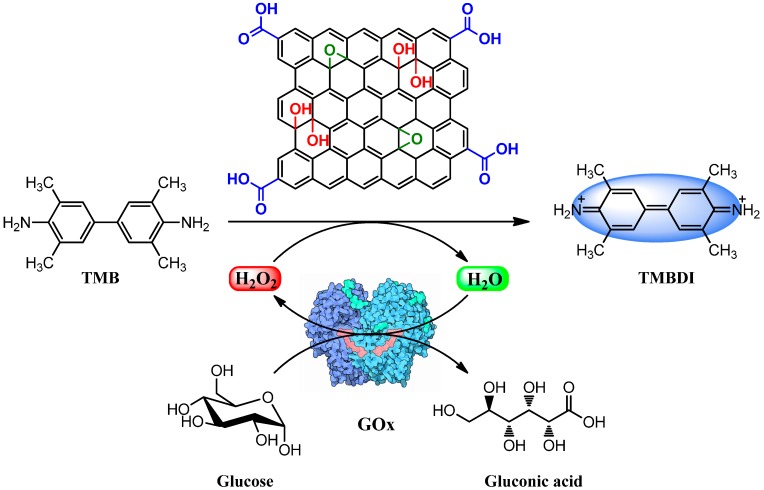
Schematic illustration of GO-catalyzed peroxidase mimic and biosensing; oxidation of TMB into TMBDI in the presence of H_2_O_2_ and colorimetric detection of glucose assisted by glucose oxidase (GOx) and GO.

By combining the GO’s activity with glucose oxidase (GOx), a simple and sensitive colorimetric assay was developed for glucose detection. In brief, the mechanism of glucose detection can be understood in two steps. Firstly, in the presence of GOx, glucose is oxidized to gluconic acid with the simultaneous conversion of substrate oxygen to H_2_O_2_. In the second step, GO reduces H_2_O_2_ in the presence of co-substrate, TMB, which itself oxidizes into TMBDI at low pH (Scheme 3). Using this assay, glucose could be detected as low as 1 × 10^−6^ mol·L^−1^ in the linear range from 1 × 10^−6^ to 2 × 10^−5^ mol·L^−1^ and thus the protocol was successfully applied to detect glucose in diluted blood, buffer solution, and commercial fruit juice samples. Interestingly, GO-based colorimetric assay gave a selective response for glucose, whereas the other glucose analogues such as fructose, maltose, and lactose remained silent. Besides glucose, the intrinsic peroxidase-like activity of GO has also been used for colorimetric detection of cancer biomarker prostate specific antigen [80].

Sun and co-workers utilized GO in a TMB-H_2_O_2_ system for the sensitive voltammetric detection of H_2_O_2_ [81]. Under the optimized conditions, the reduction peak current of the reaction product was proportional to H_2_O_2_ concentration in the linear range from 0.006 to 0.8 μmol·L^−1^ with LOD of 1.0 nmol·L^−1^. The as-proposed method was successfully applied to determine H_2_O_2_ content in fresh milk samples with recovery from 94.3% to 103.2%, demonstrating the accuracy of this method. Aside from that direct use of GO as peroxidase mimetic catalyst in biosensing, Kim, Park, and co-workers [82] utilized an indirect method to develop a GO-based immunosensing system for the detection of interleukin 5 (IL-5), a key cytokine associated with asthma pathology and eosinophilia. In particular, quenching of intrinsic GO fluorescence through HRP-catalyzed polymerization of DAB onto GO surface enabled highly sensitive sensing of IL-5 with a detection limit of *ca*. 5 pg/mL. Among other examined cytokines (IL-2, IL-6, IL-17, and interferon-γ), the GO-based immunosensing system exhibited high specificity for IL-5 and was not affected by nonspecific proteins in human serum.

Intrigued by its excellent dispersion ability for CNTs, chitosan, a biocompatible and biodegradable polymer, was employed for direct exfoliation of pristine graphite into few-layer graphene [83]. With the same concentration of 30 μg·mL^−1^, the as-prepared few-layer graphene exhibited *ca.* 45 and 4 times higher peroxidase-like activity than those of GO and rGO, respectively. The excellent catalytic performance of few-layer graphene was attributed to the fast electron transfer on its surface as evidenced by electrochemical measurements. The few-layer graphene was used to determine H_2_O_2_ concentration in three real water samples and recoveries were in the range of 100.1%–102.8%. A standard addition method was further used to confirm the feasibility of the system. The present study demonstrates the possible usage of graphene domain to activate other substrates or reagents for a task-specific purpose. In other work, mediated reduction of GO by a variety of quinone compounds such as anthraquinone-2-sulfonate, anthraquinone-2,6-disulfonate, anthraquinone-2-carboxylate, and 5-hydroxy-1,4-naphthoquinone was studied in the presence of a microbial species, namely, *Shewanella oneidensis* MR-1 [84]. The as-prepared quinone-mediated rGO was found to possess peroxidase-like activity. Table 3 summarizes the peroxidase-mimicking features of GO, rGO, or graphene.

**Table 3:** Peroxidase-like activity of graphene oxide, reduced graphene oxide, or grapheme ^a^.

Nanomaterial	Method	Substrate	LOD	Applications	Ref.
GO	Colorimetric	TMB	1.0 μM	Glucose detection	[37]
GO	Colorimetric	Hydroquinone	–	PSA detection	[80]
GO	Voltammetry	TMB	1.0 nM	H_2_O_2_ detection	[81]
GO ^b^	Fluorescence	DAB	5.0 pg/mL	IL-5	[82]
Graphene	Electrochemical	TMB	10 nM	H_2_O_2_ detection	[83]
rGO (QRGO ^c^)	Colorimetric	TMB	1.0 μM	Glucose detection	[84]

^a^ LOD, Limit of detection; TMB, 3,3′,5,5′-tetramethylbenzidine; PSA, Prostate specific antigen; DAB, 3,3′-diaminobenzidine; IL-5, Interleukin 5; ^b^ Intrinsic fluorescence of GO was used to explore a HRP-driven polymerization reaction; ^c^ RGO represents reduced graphene oxide as per ref. [84]; QRGO, Quinone-mediated reduced graphene oxide.

### 3.2. Graphene-Metalloprotein Conjugates as Peroxidase Mimetic Catalysts

**Figure 2:**
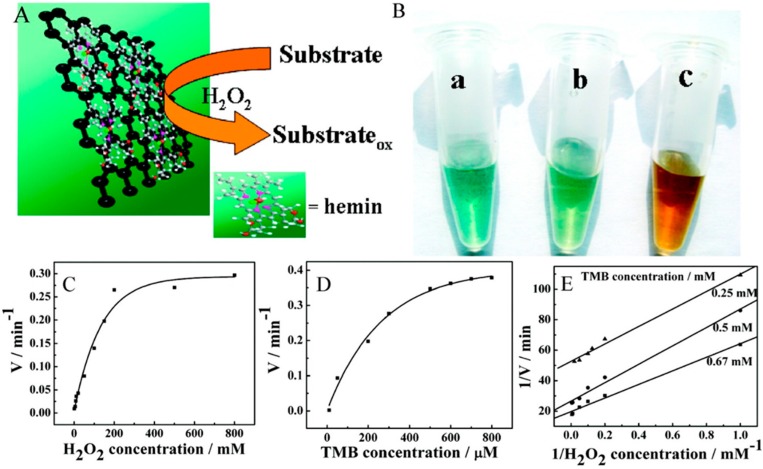
(**A**) Schematic illustration of peroxidase-like activity of H-GNs; (**B**) The H-GNs catalyze oxidation of various peroxidase substrates in the presence of H_2_O_2_ to produce different color reactions: (**a**) TMB; (**b**) ABTS; (**c**) and OPD; (**C**–**E**) Steady-state kinetic assay and catalytic mechanism of H-GNs; (**C**,**D**) The velocity (*v*) of the reaction was measured using 1 μg H-GNs in 1 mL of 25 mM PBS (pH 5.0) at room temperature; (**C**) The concentration of TMB was 0.8 mM and the H_2_O_2_ concentration was varied; (**D**) The concentration of H_2_O_2_ was 10 mM and the TMB concentration was varied; (**E**) Double-reciprocal plots of activity of H-GNs at a fixed concentration of one substrate *vs.* varying concentration of the second substrate for H_2_O_2_ and TMB. The *y*-axis values are observed absorbance values. Reproduced with permission from ref. [85]. Copyright (2011) American Chemical Society.

It is well known that hemin, an iron-protoporphyrin, is the active center of many heme-proteins such as hemoglobin, myoglobin, cytochromes, and peroxidases, capable of catalyzing a variety of oxidation reactions similar to the peroxidase enzymes. Nevertheless, its self-destruction and molecular aggregation tendency in oxidizing as well as aqueous media is the major hurdle to use it directly as an oxidation catalyst. Taking this into account, Dong and co-workers [85] relied on the use of wet-chemical strategy to synthesize hemin-graphene hybrid nanosheets (H-GNs) through π-π interactions. The as-prepared H-GNs catalyzed oxidation of TMB, ABTS, and OPD in the presence of H_2_O_2_ produced different color reactions similar to HRP. The higher peroxidase-like activity of H-GNs than that of only graphene was attributed to hemin on the graphene surface. The appearance of typical Michaelis–Menten curves (in accordance with hemin) and the apparent *K*_m_ values of H-GNs for different substrates further confirmed that H-GNs retains the peroxidase activity of hemin and the oxidation reaction follows a typical ping-pong mechanism (Figure 2). Interestingly, at optimum electrolyte concentration, H-GNs exhibited the unique ability of differentiating ss- and ds-DNA, and this feature was successfully used in the naked-eye colorimetric detection of single-nucleotide polymorphisms (SNPs) in disease-associated DNA at room temperature [85].

Based on the synergistic features of H-GNs, the group further reported highly sensitive dual biosensor (amperometric as well as colorimetric) for the detection of H_2_O_2_ and glucose [86]. In other work, a folic acid-conjugated graphene-hemin composite (GFH) with peroxidase-like activity was developed for rapid and quantitative colorimetric detection of as low as 1000 cancer cells [87]. Notably, the detection assay also demonstrated the specificity of GFH for HeLa and MCF-7 over NIH-3T3 cells. Compared to other systems such as mesoporous silica and CNTs, the GFH exhibited good stability and robustness.

**Scheme 4:**
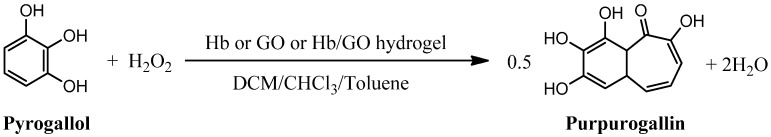
Schematic of Hb or GO or Hb/GO hydrogel catalyzed oxidation of pyrogallol with H_2_O_2_ in the presence of different organic solvents.

Considering the pilot role of hydrogels as excellent support for immobilization of enzymes, Shi and co-workers [88] developed a supramolecular hydrogel to study a peroxidation reaction in organic solvents. Typically, hydrogel was prepared by direct mixing the dispersions of GO and hemoglobin (Hb) in DI water through non-covalent interactions. The catalytic activity of as-prepared GO/Hb hydrogel was tested for oxidation of pyrogallol in the presence of H_2_O_2_ (Scheme 4). Interestingly, the GO/Hb hydrogel exhibited greater stability and higher average activity than that of free Hb or GO in polar to non-polar organic solvents. The higher activity may be attributed to the aqueous microenvironment of hydrogel that protects the enzyme from deactivation and also serves as a transit station for substrate and product simultaneously. The ease of synthesis and preserving its catalytic activity even after storing at room temperature for a long duration are the attractiveness of GO-based hydrogel in peroxidase mimic.

The oxidation of pyrogallol into purpurogallin was also studied using hemin-graphene conjugate as a highly active biomimetic catalyst in conjunction with an immobilized iron-porphyrin derivative, iron(III)meso-tetrakis(*N*-methylpyridinium-4-yl)porphyrin (FeTMPyP), onto graphene surface [89]. The spectroscopic studies revealed the formation of an axial ligation to the iron center of hemin via cation-π interactions between iron centers and graphene in which hemin retains its active monomeric form, similar to natural enzymes. The kinetic studies revealed that the catalytic efficiencies of hemin-graphene (*K*_cat_/*K*_M_ ~ 2 × 10^5^ M^−1^·min^−1^) and FeTMPyP-graphene (*K*_cat_/*K*_M_ ~ 5.7 × 10^5^ M^−1^·min^−1^) conjugates were *ca.* 100 times more than that of free or dimeric hemin and approaching to that of the native HRP (*K*_cat_/*K*_M_ ~ 2 × 10^6^ M^−1^·min^−1^). It is worth noting that graphene as a support not only prevents the hemin from either self-dimerization or oxidative destruction but also provides relatively large surface area, leading to the higher reaction turnover rate due to strong binding interactions. Very recently, the peroxidase-like activity of hemin-graphene conjugates has been used to develop a label-free colorimetric biosensing assay for DNA and cocaine, one of the most dangerous illegally abused drugs to human health [90]. The kinetic studies revealed that the biosensor had good selectivity for cocaine among others typical interfering compounds including caffeine, theophylline, and morphine hydrochloride.

**Figure 3:**
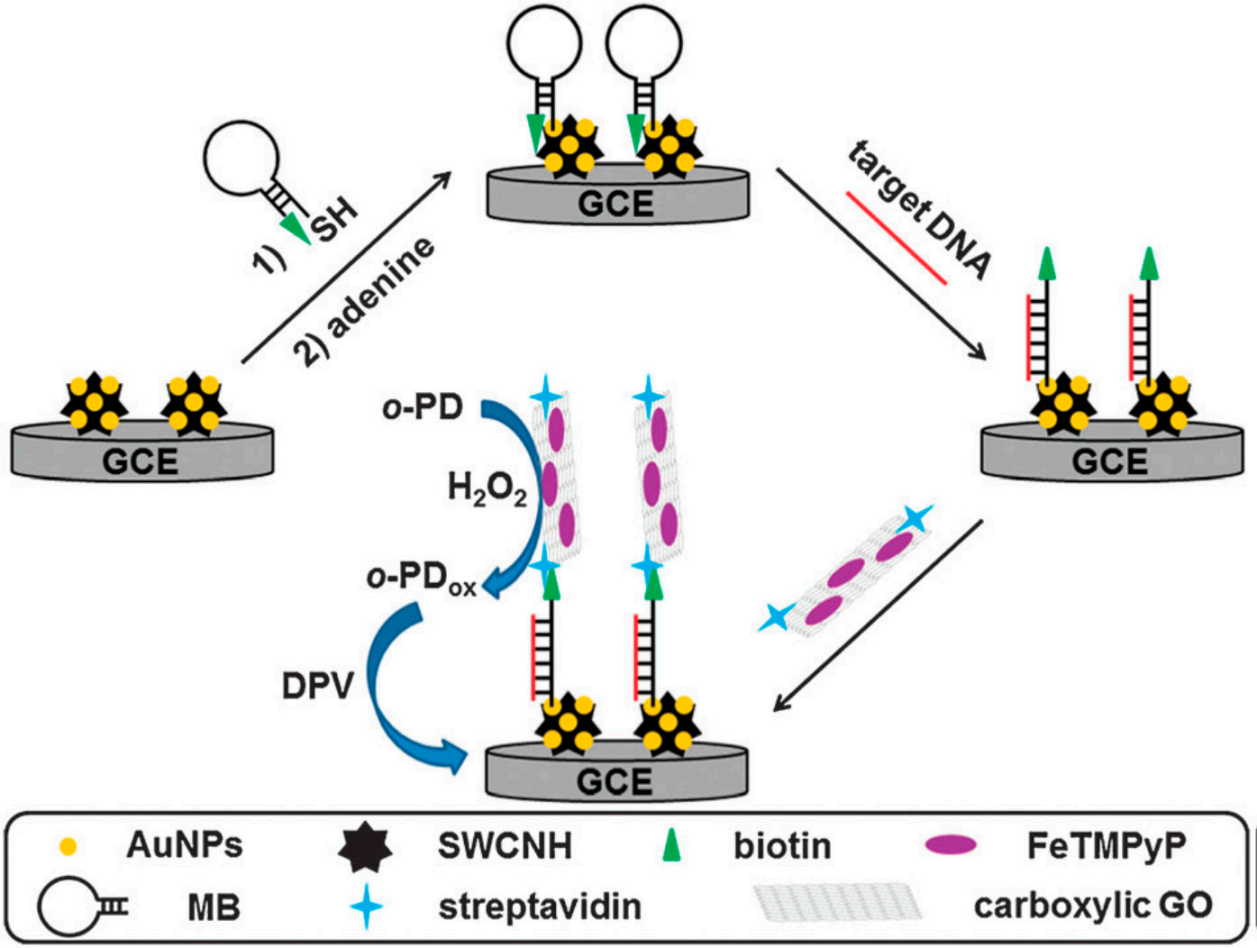
Schematic illustration of graphene-supported ferric porphyrin as a HRP mimicking trace label for electrochemical detection of DNA. Reproduced with permission from ref. [91]. Copyright (2012) Royal Society of Chemistry. Abbreviations: GCE, Glassy carbon electrode; MB, Molecular beacon; FeTMPyP, Iron(III)meso-tetrakis(*N*-methylpyridinium-4-yl)porphyrin; SWCNH, Single-walled carbon nanohorn; *o*-PD, Ortho-phenylenediamine; GO, Graphene oxide.

Ju and co-workers [91] fabricated a universal peroxidase mimic by loading FeTMPyP onto GO. The integration of FeTMPyP-GO with streptavidin by an amidation reaction resulted in the formation of FeTMPyP-streptavidin-GO bioconjugate, which exhibited an enhanced peroxidase-like activity toward OPD oxidation in the presence of H_2_O_2_. By combing this trace label with a biotinylated molecular beacon (MB) immobilized on an AuNPs-single-walled carbon nanohorn (AuNPs-SWCNH) modified glassy carbon electrode (GCE), a sensitive “signal on” biosensor was developed for electrochemical detection of DNA down to attomolar levels (Figure 3). The differential pulse voltammetric (DPV) measurements suggested that the peroxidase-like activity of FeTMPyP- streptavidin-GO modified GCE was generated from the integration of GO and FeTMPyP. In this way, the porphyrin-based trace label is highly promising in mimicking natural enzymes.

**Figure 4:**
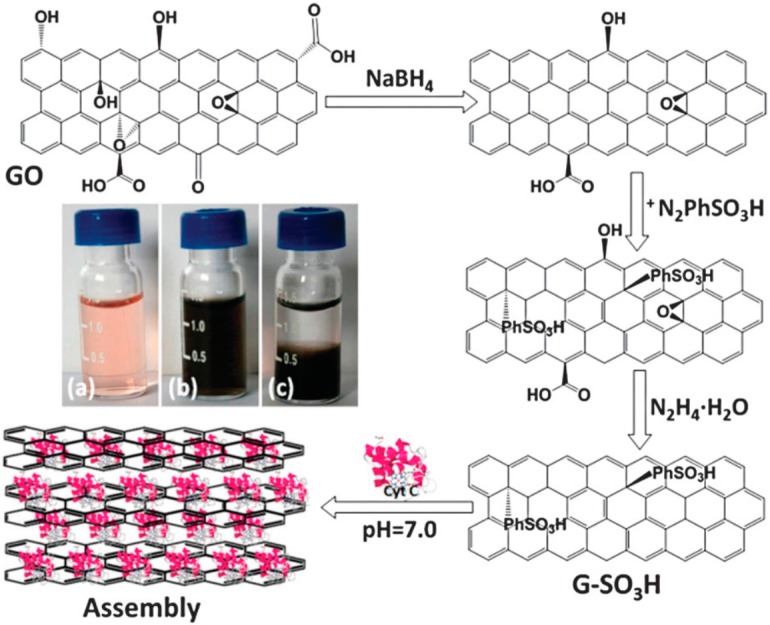
Illustration of the synthesis of G-SO_3_H and its assembly with cyt C. Inserted photos: (**a**) cyt C solution; (**b**) fresh mixture solution of cyt C and G-SO_3_H; (**c**) colorless supernatant and sediment after assembling for 24 h. Reprinted with permission from ref. [92]. Copyright (2012) Royal Society of Chemistry.

Xia and co-workers [92] studied the catalytic and electrochemical behavior of cytochrome C (cyt C), an electron transfer protein in the respiratory chain, confined in layered nanospace by assembling it within covalently modified sulfonated graphene (G-SO_3_H) nanosheets using electrostatic interactions (Figure 4).

In a confined environment, the G-SO_3_H/cyt C assembly could catalyze the oxidation of OPD, similar to free cyt C, whereas G-SO_3_H alone exhibited extremely low catalytic activity. The kinetic studies further revealed that despite having limited substrate diffusion in confined nanospace, the G-SO_3_H/cyt C assembly exhibits higher catalytic activity (*K*_m_ 31.3 mM) than free cyt C (*K*_m_ 235 mM). It can be attributed to the higher probability of substrate to collide with cyt C, which, otherwise, is limited or absent in the native cyt C. This work sheds light on the fact that the property of proteins in the confined environments may differ significantly from their free states and thus the native properties of proteins can be fine-tuned using engineered nanostructures like graphene. Table 4 summarizes the notable features of graphene-metalloprotein conjugates being used as efficient peroxidase mimics for biosensing applications.

**Table 4:** Peroxidase-like activity of graphene-metalloprotein conjugates ^a^.

Nanomaterial	Method	Substrate	LOD	Applications	Ref.
H-GNs	Colorimetric	TMB, ABTS, and OPD	–	SNPs detection	[85]
H-GNs		TMB	0.2 μM	H_2_O_2_ detection	[86]
	Amperometric		0.3 μM	Glucose detection	
	Colorimetric		20 nM	H_2_O_2_ detection	
			30 nM	Glucose detection	
GFH	Colorimetric	TMB	1000 CC	CC detection	[87]
GO/Hb hydrogel	–	Pyrogallol	–	–	[88]
H-GCs	–	Pyrogallol	–	–	[89]
FeTMPyP-GCs					
H-GNs	Colorimetric	ABTS, TMB, and OPD	9 nM	DNA detection	[90]
			20 nM	Cocaine detection	
FeTMPyP- streptavidin-GO BCs	Electrochemical	OPD	22 aM	DNA detection	[91]
G-SO_3_H/cyt C Ns	Electrochemical	OPD	–	–	[92]
DNA-H-GNs	Colorimetric	TMB	8 nM	Hg^2+^ detection	[93]
			0.5 nM	DNA detection	
DNA-H-GNs	Colorimetric	TMB		Protein detection:	[94]
			0.5 nM	Thrombin	
			5 nM	PDGF-BB	
H-GNs	Electrochemical	HQ	0.17 pM	microRNAs detection	[95]
H-GNs	Colorimetric	4-AAP	–	Phenol detection	[96]

^a^ H-GNs, Hemin-graphene hybrid nanosheets; SNPs, single-nucleotide polymorphisms; GFH, Folic acid conjugated graphene-hemin composite; CC, Cancer cells; Hb, Hemoglobin; Cs, Conjugates; H-GCs, Hemin-graphene conjugates; FeTMPyP, iron(III)meso-tetrakis(*N*-methylpyridinium-4-yl)porphyrin; BCs, Bioconjugates; aM, Attomolar (10^−18^ M); cyt C, Cytochrome C; Ns, Nanosheets; PDGF-BB, Platelet-derived growth factor; HQ, Hydroquinone; 4-AAP, 4-aminoatipyrene.

### 3.3. Graphene-Gold Hybrid Nanostructures as Peroxidase Mimetic Catalysts

**Figure 5:**
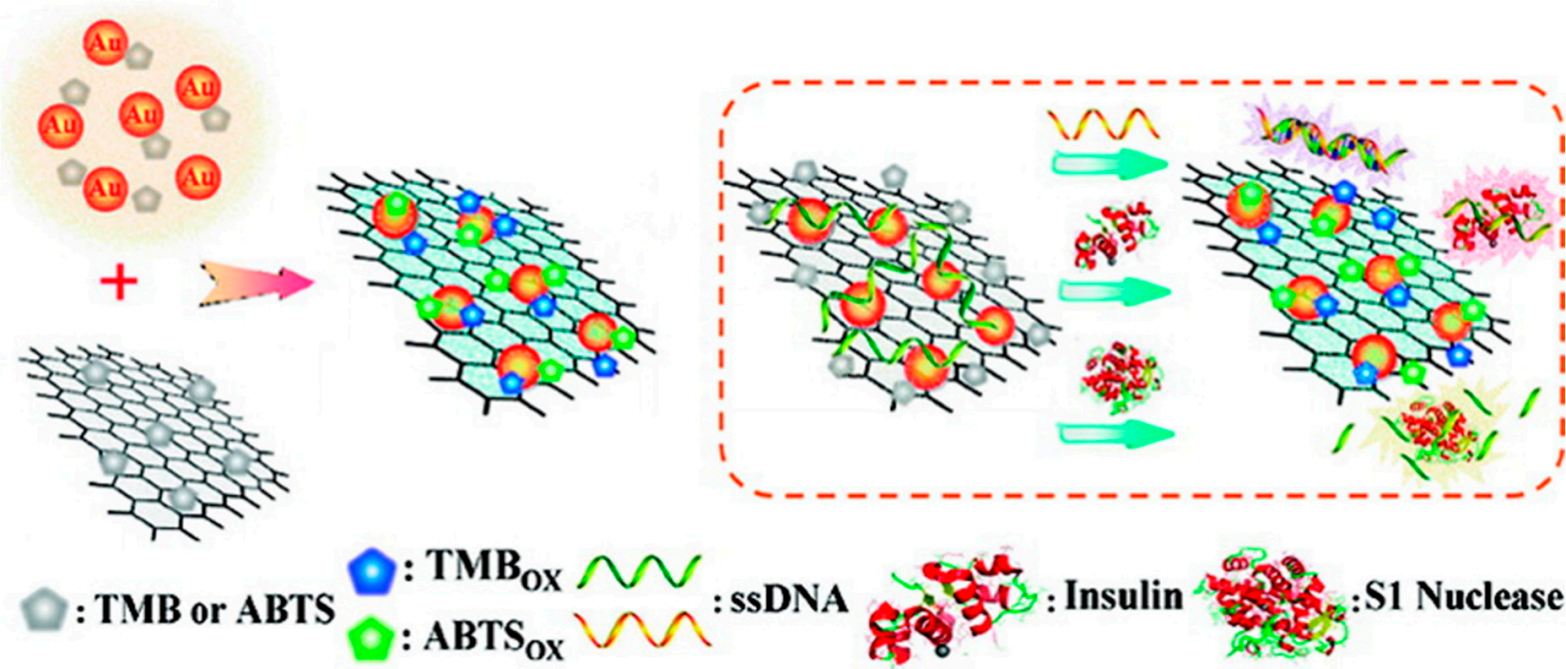
Schematic illustration of a versatile and label-free colorimetric biosensing platform based on the tunable smart interface of graphene-AuNPs hybrid catalyst. Reproduced with permission from ref. [98]. Copyright (2012) American Chemical Society.

Since its first appearance in 2004 [24], nano-gold of varying sizes and shapes has become a thriving material of scientific interest, especially, in enzyme mimics [39,97]. In this context, a new generation of G-NMs is particularly impressive and offers great opportunities in catalytic processes at bio-nano interface. For instance, Quan and co-workers [98,99] developed a hybrid catalyst by growing AuNPs on graphene sheets *in situ*. The as-obtained graphene-AuNPs hybrid exhibited high peroxidase-like activity than both either bare AuNPs or graphene alone, which may be attributed to the synergetic coupling effect between the two components having very little activity. Interestingly, the interface of hybrid catalyst could be reversibly switched from passive to active based on the physical adsorption and responsive detachment of ssDNA from the interface, respectively [98]. Such a reversible switching interface in graphene-AuNPs hybrid prevents the specific sites of peroxidase substrates from diffusing and binding, thus serves as a versatile basis for designing label-free colorimetric biosensors, DNA, in particular with little or no background signals (Figure 5).

**Figure 6:**
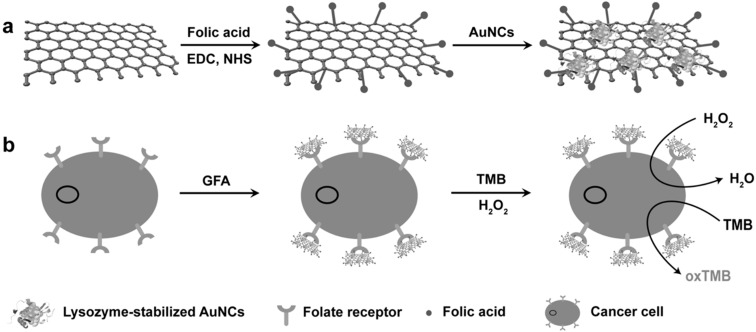
Schematic representation of (**a**) preparation of FA-GO-AuNCs hybrid (GFA) and (**b**) cancer cell detection by using target-directed GFA. Reproduced with permission from ref. [100]. Copyright (2013) Wiley-VCH Verlag GmbH & Co. Abbreviations: EDC, 1-Ethyl-3-(3-dimethylaminopropyl)carbodiimide; NHS, *N*-hydroxysulfosuccinimide; AuNCs, Gold nanoclusters; GFA, Folic acid-graphene oxide-AuNCs hybrid.

Similar synergetic effect was also realized with GO-Au hybrid nanoclusters (GO-AuNCs) that can work over a wide pH range [100], a feature that was not previously accessible with either GO alone or Au-nanostructures under virtually identical conditions. Given the high surface-to-volume ratio as well as high affinity of GO for hydrophobic molecules, the remarkable enhancement in the peroxidase-like activity of GO-AuNCs hybrid, may be attributed to the key role of GO as an efficient modulator, bringing substrate such as TMB into proximity of the active sites of AuNCs, thus enhancing the catalytic activity of GO-AuNCs hybrid in a fairly broad range of operating pH. Moreover, upon conjugation of folic acid to the GO-AuNCs hybrid, a selective, quantitative, and robust nanoprobe, FA-GO-AuNCs hybrid (GFA), could be developed for colorimetric detection of as low as 1000 cancer cells (Figure 6). It would be worthwhile to mention here that the specificity of GFA for cancer cells was determined to be in the following order: MCF-7 cells > HeLa cells > NIH-3T3 cells, similar to that what observed with GFH [87].

**Figure 7:**
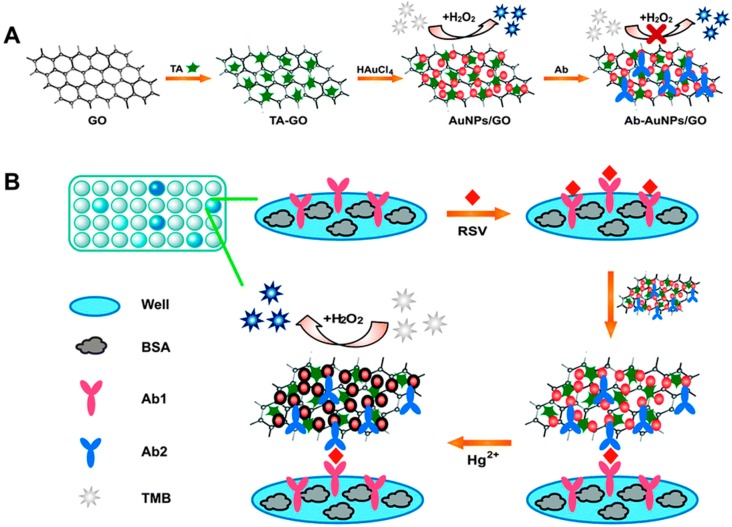
(**A**) Procedure for the preparation of Ab-AuNPs-GO conjugates; (**B**) A schematic representation of the Hg^2+^-enhanced peroxidase-like activity of AuNPs-GO hybrids for colorimetric immunoassays of RSV. Reproduced with permission from ref. [101]. Copyright (2014) Royal Society of Chemistry.

Intrigued by mercury ion (Hg^2+^)-enhanced enzymatic activity of citrate-capped AuNPs, Huang and co-workers [101] have also made use of this feature to AuNPs-GO hybrids, which was prepared by the added advantages of tannic acid (TA), a water soluble polyphenolic compound, being used both as reducing and stabilizing agent. The as-prepared AuNPs-GO hybrids produced a typical color change due to the oxidation of TMB in the presence of H_2_O_2_. However, the peroxidase-like catalytic activity of AuNPs-GO hybrids significantly decreased after their binding with antibodies (Ab) as shown in Figure 7A. Upon the addition of Hg^2+^, the catalytic activity of AuNPs-GO was stimulated, even in the presence of protein. The Hg^2+^-stimulated catalytic ability of Ab-AuNPs-GO conjugates was found to be much stronger due to the high-specificity metallophilic Hg^2+^–Au interactions, inducing the deposition of Hg atoms onto the surfaces of AuNPs. Furthermore, the high activity of Ab-AuNPs-GO conjugates relative to those of both Ab-GO and Ab-AuNPs may be attributed to the synergetic coupling effects between AuNPs and GO as demonstrated previously [98,99,100]. The Hg^2+^-triggered peroxidase-like activity of AuNPs-GO hybrids was successfully applied in a sandwich-based colorimetric immunoassay for highly sensitive detection of respiratory syncytial virus (RSV), one of the major causes for respiratory tract infections (Figure 7B).

Given the excellent catalytic performances based on the synergistic interactions between individual components, recently, a good progress has been made (and will continue to be) to synthesize graphene-Au hybrid NMs with peroxidase-like activities that hold great promises in biochemistry, biotechnology, and biomedical sciences in near future. The notable features of this class of G-NMs with peroxidase-like activities are summarized in Table 5.

**Table 5:** Peroxidase-like activity of graphene-Au hybrid NMs ^a^.

Nanomaterial	Method	Substrate	LOD	Applications	Ref.
G-AuNPs hybrid	Colorimetric	TMB, ABTS, and OPD	–	DNA detection	[98,99]
(FA)-GO-AuNCs	Colorimetric	TMB	1000 CC	CC detection	[100]
GO-AuNPs hybrid	Colorimetric	TMB	0.04 pg/mL	RSV detection	[101]
H-RGO-Au composite	Colorimetric	TMB	5 nM	H_2_O_2_ detection	[102]
AuNPs/Cit-GNs composite	–	TMB, ABTS	–	–	[103]
GSHA hybrid *^b^*	–	TMB	–	–	[104]
G-AuNPs hybrid	Colorimetric	TMB	0.0016 U/μL	hOGG1 detection	[105]
GO-AuNPs hybrid	Colorimetric	TMB	0–50 μM	Hg^2+^ and Pb^2+^ detection	[106]
GSF@AuNPs hybrid	Colorimetric	TMB	50 CC	CC detection	[107]
Au-rGO composite	Colorimetric	Pyrogallol	–	Dye removal	[108]

^a^ G, Graphene; FA, Folic acid; AuNCs, Gold nanoclusters; CC, Cancer cells; RSV, Respiratory syncytial virus; H-RGO-Au, Hemin-reduced graphene oxide-gold; Cit-GNs, Citrate-functionalized graphene nanosheets; GS, Graphene-mesoporous silica hybrid; GSHA, Graphene-mesoporous silica-hemin-gold nanoparticles hybrid; hOGG1, Human 8-hydroxyguanine glycosylase, one type of DNA glycosylase expressed in human tissues; GSF, Folic acid-conjugated PMS coated RGO; PMS, Periodic mesoporous silica; RGO, Reduced graphene oxide as per refs. [102,107]. *^b^* The catalyst can also function in oxidase mimic.

### 3.4. Graphene-Fe_x_O_y_ Magnetic Nanocomposites as Peroxidase Mimetic Catalysts

**Figure 8:**
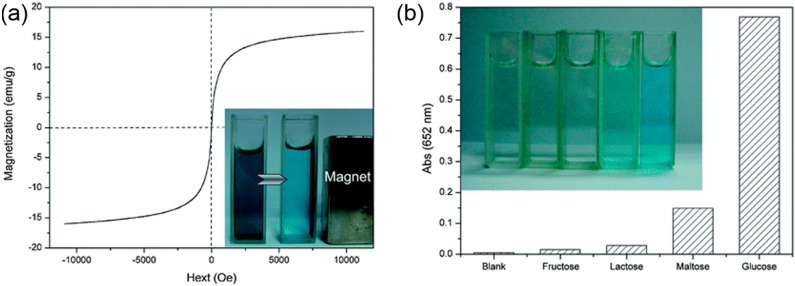
(**a**) Magnetization curve of GO-Fe_3_O_4_ nanocomposites. Inset: Image of solution before and after magnetic separation of GO-Fe_3_O_4_ nanocomposites. (**b**) Selectivity analysis for glucose detection by monitoring the absorbance at 652 nm. The analyte concentrations were as follows: 5 mM lactose, 5 mM fructose, 5 mM maltose, and 0.5 mM glucose. Inset: The color change for the different solutions. Reproduced with permission from ref. [109]. Copyright (2012) Royal Society of Chemistry.

Magnetic nanoscale structures such as iron oxide (magnetite or Fe_3_O_4_) exhibit distinct potential in biochemical and industrial fields. Based on its mixed-valence feature, Fe_3_O_4_ is also known as binary iron oxide or ferrous-ferric oxide. Indeed, the presence of ferrous (Fe(II)) and ferric ions (Fe(III)) in Fe_3_O_4_ NPs has been demonstrated as the key to their peroxidase-like activity [8]. Given the remarkable stability of Fe_3_O_4_ NPs over a wide range of temperature and pH (1–12), Chen and co-workers [109] prepared GO-Fe_3_O_4_ nanocomposites by dropwise addition of tris(acetylacetonato)iron(III), (Fe(acac)_3_), dispersed in 1-methyl-2-pyrrolidne, to the GO suspension. The superparamagnetic behavior of as-prepared GO-Fe_3_O_4_ was realized based on the S-like appearance of magnetization hysteresis loop as well as nearly zero magnetic remanence. Furthermore, the saturation magnetization value of 16 emu·g^−^^1^ clearly indicated that the GO-Fe_3_O_4_ can be quickly separated from the solution just by making the use of an external magnet (Figure 8a). The peroxidase-like activity of GO-Fe_3_O_4_ was demonstrated by oxidation of TMB in the presence of H_2_O_2_, producing a blue color. The kinetic studies indicated that the catalytic behavior of GO-Fe_3_O_4_ follows a typical ping-pong mechanism [109]. Based on the intrinsic peroxidase-like activity of GO-Fe_3_O_4_ nanocomposites, a colorimetric assay was developed for the detection of H_2_O_2_ and glucose. Notably, the nanocomposites exhibited a glucose selective color response even in the presence of higher concentrations of other glucose analogues like fructose, lactose, and maltose (Figure 8b). Furthermore, the practical applicability of method was explored by detecting glucose in urine of a diabetic person that gave recoveries from 95.1% to 104.1%. The ease of synthesis, handy separation, cost-effectiveness, remarkable affinity due to synergistic features of both GO and Fe_3_O_4_ NPs, and superparamagnetic properties of GO-Fe_3_O_4_ nanocomposites make it a material of choice in biochemical/medical as well as industrial fields.

Intrigued by the considerable effect of sizes and shapes on the peroxidase-like activity of Fe_3_O_4_ NPs, Wang and co-workers [110] prepared Fe_3_O_4_ nanospheres/rGO (Fe_3_O_4_ NSs/rGO) composite following a solvothermal method. Compared with Fe_3_O_4_ microspheres (MSs)/rGO and Fe_3_O_4_ nano-polyhedrons (NPHs)/rGO, the resultant Fe_3_O_4_ NSs/rGO nanocomposites exhibited highest peroxidase-like activity as evidenced by oxidation of TMB. Based on this specific activity of Fe_3_O_4_ NSs/rGO nanocomposites, a facile and sensitive method was developed for the colorimetric detection of acetylcholine (Ach), an important signal transmission molecule in the central nervous system. The nanocomposite exhibited a fairly selective response towards Ach while the absorbances from other interfering substances such as ascorbic acid (AA), cysteine (Cys), and aspartate were negligible for Ach.

This work sheds light on the fact that the selective or controllable fabrication of graphene-magnetic nanocomposites with desired morphologies can play a key role in peroxidase mimics. This has been demonstrated with cubic shaped Fe_3_O_4_ NPs loaded on GO-dispersed CNTs [111] as well as *in situ* growth of cubic CoFe_2_O_4_ ferrite on graphene surface [112].

**Figure 9:**
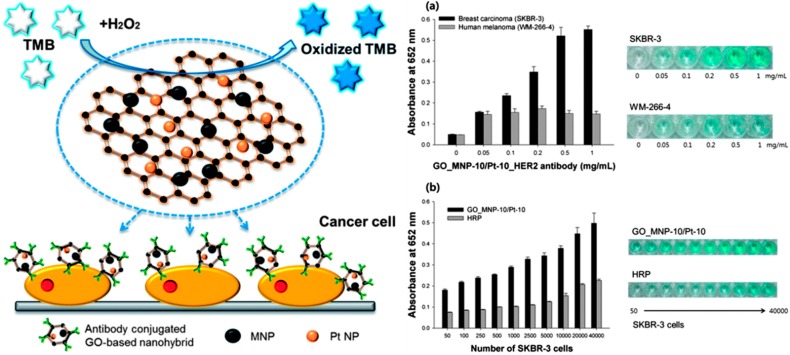
**Left panel**: Schematic representation of colorimetric detection of target cancer cells based on the hybrid nanocomposite entrapping both MNPs and Pt NPs on carboxyl-modified graphene oxide; **Right panel**: Absorption intensities of the blue color signal and their corresponding well plate images to (**a**) specifically detect SKBR-3 cells by using a GO_MNP-10-Pt-10 nanohybrid and; (**b**) quantitatively detect SKBR-3 cells by using GO_MNP-10-Pt-10 nanohybrid and HRP. The error bars represent the standard deviation of three independent measurements. Reproduced with permission from ref. [114]. Copyright (2013) Royal Society of Chemistry.

Taking into account the synergistic effect with enhanced catalytic activities of both Fe_3_O_4_ NPs (MNP) and Pt NPs in oxygen reduction reaction [113], a new nanocomposite was prepared by immobilizing catalytically active MNP and Pt NPs simultaneously onto GO surface [114]. The peroxidase-like activity of the as-obtained hybrid composite, GO_MNP-10-Pt-10 (the number represent the weight % of NPs), in particular was demonstrated in conjunction with bare GO, free Fe_3_O_4_ NPs, free Pt NPs, and other GO-entrapping metal NPs (GO-Pt-10, GO_MNP-10/30, and GO_MNP-30-Pt-10) using TMB as a substrate. Among all tested catalysts, the GO_MNP-10-Pt-10 exhibited best volumetric activity (ν_max_ 2180.9 nM·S^−1^) after bare Pt NPs (ν_max_ 4002.2 nM·S^−1^) leading to the formation of deep blue color of oxidized TMB. Furthermore, the steady state kinetic measurements suggested that the immobilization of both MNP and Pt NPs onto GO surface substantially relieves the mass transfer limitations (of both types of NPs) by the affirmative effect of GO. The peroxidase-like activity of GO_MNP-10-Pt-10 was used to develop a sandwich-type immunoassay for colorimetric detection of target cancer cells (Left panel: Figure 9). In detail, GO_MNP-10-Pt-10 hybrid nanocomposite was conjugated with antibodies directed against human epidermal growth factor receptor 2 (HER2) using the anchored –COOH groups on GO surface. The resultant antibody-conjugated GO_MNP-10-Pt-10 was used to detect Human breast adenocarcinoma cells (SKBR-3); whereas Human melanoma cells (WM-266-4), which lack HER2 expression, were employed as negative control. As shown in Figure 9a (right panel), the wells containing only SKBR-3 cells produced the specific color modulations within 5 min, validating the specific binding of nanocomposite to SKBR-3 cells. Furthermore, as the amount of the cells increased, the antibody-conjugated GO_MNP-10-Pt-10 nanocomposite showed significantly higher level of absorption intensity than that of native HRP with LOD as low as 100 SKBR-3 cells in the linear range from 100–1000 cells (Right panel: Figure 9b). The present protocol using GO-based synergistic nanocomposite offers an efficient point-of-care detection in clinical diagnostics.

Recently, magnetic G-NMs have become a burgeoning area of scientific interests, featuring the multi-functions of remarkably high peroxidase-like catalysis, which otherwise was not seen previously with bare inorganic catalysts (Table 6).

**Table 6:** Peroxidase-like activity of graphene-based magnetic NMs ^a^.

Nanomaterial	Method	Substrate	LOD	Applications	Ref.
GO-Fe_3_O_4_ composite	Colorimetric	TMB	0.32 μM	H_2_O_2_ detection	[109]
			0.74 μM	Glucose detection	
Fe_3_O_4_ NSs-rGO composite	Colorimetric	TMB	39 nM	Ach detection	[110]
GCNT-Fe_3_O_4_ composite	Colorimetric		–	H_2_O_2_ detection	[111]
		TMB, OPD, DAB, PAP, and HQ	–	Glucose detection	
		TMB			
	Electrochemical		22 μM	Glucose detection	
rGO-CF composite	Colorimetric	TMB	0.3 μM	H_2_O_2_ detection	[112]
GO_MNP-10-Pt-10 composite	Colorimetric	TMB	100 CC	CC detection	[114]
mFe_2_O_3_-G composite	Colorimetric	TMB	0.5 μM	Glucose detection	[115]
MNP-GO-H composite	Colorimetric	ABTS	0.08 nM	GSH detection	[116]
AR/FeO_x_H-rGO composite	Fluorescence	AR	50 nM	H_2_O_2_ detection	[117]
			50 nM	S^2–^ detection	
3DRGO_ Fe_3_O_4_-Pd composite	Colorimetric	TMB	86 nM	H_2_O_2_ detection	[118]
			52 nM	GSH detection	
			0.13 μM	Glucose detection	
RGO-INs composite	Colorimetric	TMB, OPD, and THB	0.2 μM	H_2_O_2_ detection	[119]
			0.8 μM	Glucose detection	
Hg^2+^/Au-Fe_3_O_4_-GO composite	Colorimetric	TMB	0.15 nM	Hg^2+^ detection	[120]
			>96%	Hg^2+^ removal	

^a^ NSs, Nanospheres; Ach, Acetyl choline; GCNT, GO-dispersed carbon nanotubes; PAP, *p*-amino phenol; rGO, Reduced graphene oxide; DAB, 3,3′diaminobenzidine; CF, CoFe_2_O_4_ ferrite; MNP, Magnetic nanoparticles; CC, Cancer cells; mFe_2_O_3_-G, Mesoporous Fe_2_O_3_ graphene; MNP-GO-H, Magnetic nanoparticle-GO supported hemin; GSH, Glutathione; AR, Amplex red (10-acetyl-3,7-dihydroxyphenoxazine); FeO_x_H-rGO, Amorphous iron hydroxide/oxide immobilized on rGO; S^2−^, Sulfide anion; THB, 1,2,3-trihydroxybenzene; 3DRGO; Three dimensional reduced graphene oxide as per ref. [118]; RGO-IN, reduced graphene oxide-iron nanoparticles as per ref. [120].

### 3.5. Graphene-Based other NMs as Peroxidase Mimetic Catalysts

Aside from those categorized above, this section deals with most notable advancements in peroxidase mimics within the framework of G-NMs. Huang and co-workers [121] prepared a rGO-supported Co_3_O_4_ composite by *in situ* controlled nucleation of Co_3_O_4_ NPs onto GO nanosheets and subsequent *in situ* reduction of GO following hydrothermal reaction in ethanol media at low temperature. The peroxidase-mimicking activity of rGO-Co_3_O_4_ was confirmed by catalytic oxidation of TMB. Kinetic studies showed that the nanocomposite had higher affinity towards TMB (*K*_m_ 0.19 mM) but lower affinity towards H_2_O_2_ (*K*_m_ 24.04 mM) when compared with HRP (TMB: *K*_m_ 0.434 mM, H_2_O_2_: *K*_m_ 3.70 mM). The mechanistic studies further suggested that the peroxidase-like activity was due to nanocomposite’s ability of accelerating the electron-transfer process and the consequent facilitation of radical generation. The rGO-Co_3_O_4_ composite was successfully used in the selective colorimetric detection of glucose over other analogues such as fructose, lactose, and maltose.

**Figure 10:**
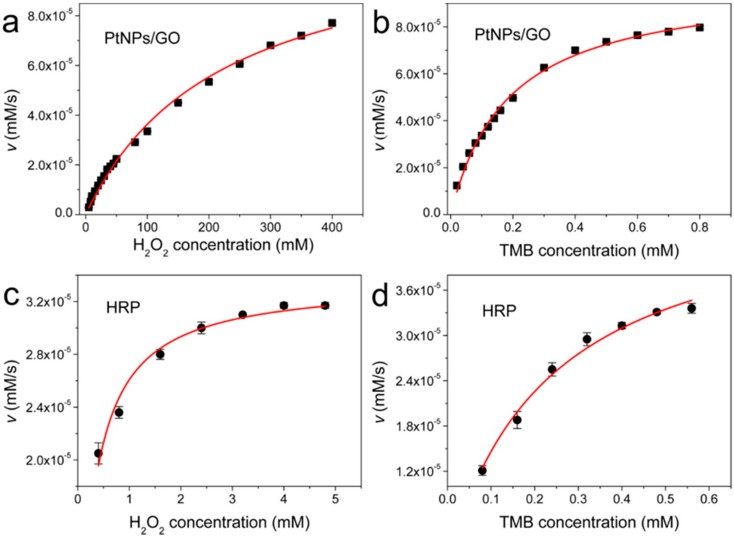
Steady state kinetic assay of Pt NPs-GO composite and HRP using 20 mM phosphate buffer (pH 5.0) at 30 °C. (**a**,**c**) TMB concentration was fixed at 0.8 mM, and H_2_O_2_ concentration was varied; (**b**,**d**) H_2_O_2_ concentration was fixed at 400 mM for Pt NPs-GO composite or 5 mM for HRP, and the TMB concentration was varied. Reprinted with permission from ref. [122]. Copyright (2014) American Chemical Society.

In another study, GO was used both as stabilizer and reductant to obtain stable, clean, and well-dispersed porous Pt NPs on its surface [122]. The as-obtained porous Pt NPs-GO nanocomposite could catalyze the oxidation of TMB in the presence of H_2_O_2_ and the catalytic activity was found to be dependent on pH, temperature, and concentration, similar to those of HRP and other NMs-based peroxidase mimics. The *K*_m_ from the Lineweaver–Burk plot (Figure 10) showed that the porous Pt NPs-GO had less affinity to H_2_O_2_ compared with HRP (221.4 mM for Pt NPs-GO *vs.*0.2832 mM for HRP), but had higher affinity to TMB compared with HRP (0.1864 mM for Pt NPs-GO *vs.*0.2343 mM for HRP). Based on its peroxidase-like activity, the folic acid-conjugated porous Pt NPs-GO was used for colorimetric detection of cancer cells.

In their follow-up work [123], the group further anchored Pt NPs onto GO surface using borohydride reduction method for efficient peroxidase mimic. The steady state kinetic assay indicated that as-obtained Pt NPs-GO nanocomposite had highest affinity to both TMB and H_2_O_2_ compared with other types of Pt NPs such as ferritin-Pt NPs, citrate-PtNPs, and porous Pt NPs-GO nanocomposite. Furthermore, a higher affinity of nanocomposite towards TMB (0.041 mM) over HRP (~0.2 mM) demonstrates the significant advantage of G-Pt NPs-based peroxidase mimics. Based on the target-induced inhibition of the peroxidase-like activity of Pt NPs-GO nanocomposite, a colorimetric assay was developed for the sensitive and selective detection of cysteine, especially, at lower concentration. However, at concentrations higher than 0.5 μM, slight interference was recognized in the presence of methionine and structurally similar glutathione.

In Chen and Su’s work [124], bimetallic PtPd nanodendrites (NDs) composite was synthesized on graphene nanosheets (GNs) by the reduction of ethanol. The as-obtained PtPdNDs-GNs were found to have intrinsic peroxidase-like activity and the catalysis was strongly dependent on pH and temperature, following a ping-pong mechanism. A stronger affinity towards TMB and a higher catalytic activity (lower value of *K*_m_) of PtPdNDs-GNs than those of Pt nanoflowers-GNs, Pt NPs-GNs, bimetallic PtPd nanoalloys-GNs, and core-shell Pd@Pt nanoflowers-GNs was attributed to the bimetallic composition as well as NDs morphology. Very recently, a dual functional, Pt-on-Pd-rGO nanocomposite is reported [125]. Owing to the synergistic functions between Pt-on-Pd and rGO, the nanocomposite exhibits superior peroxidase-like activities and an enhanced electrocatalytic oxidation feature.

**Figure 11:**
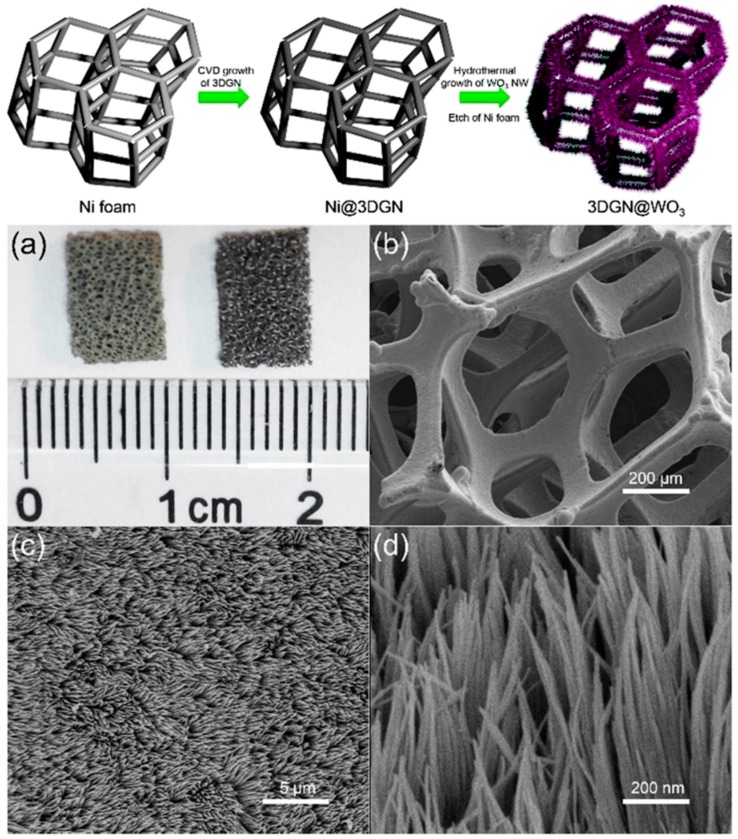
Upper panel: synthesis process of 3DGN@WO_3_ NW arrays; Lower panel: morphology of 3DGN@WO_3_. (**a**) Digital image of 3DGN@WO_3_ (**left**) and 3DGN (**right**); (**b**–**d**) Low to high magnification SEM images of 3DGN@WO_3_. Reproduced with permission from ref. [126]. Copyright (2014) Royal Society of Chemistry.

**Figure 12:**
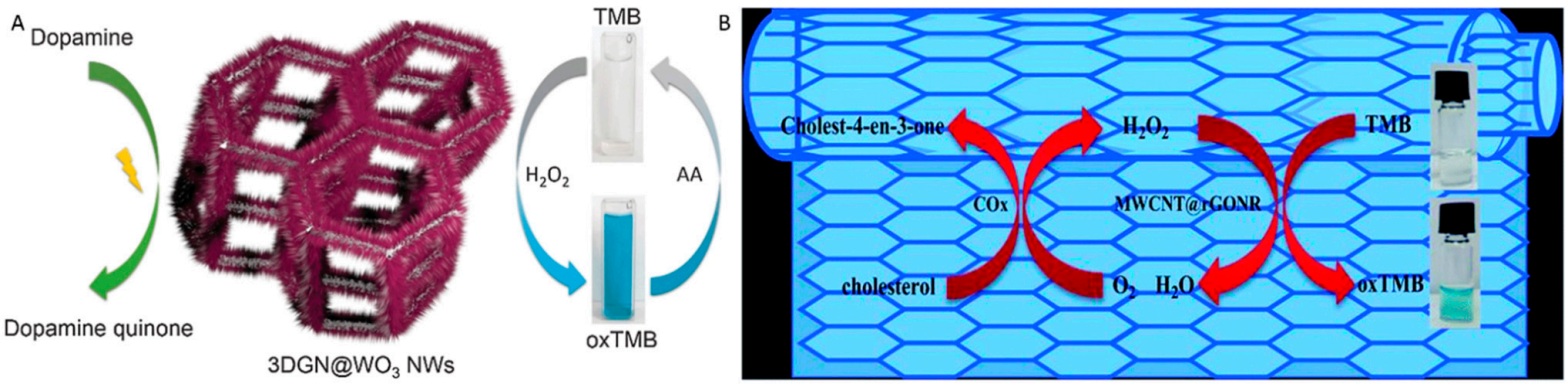
(**A**) Schematic representation of 3DGN@WO_3_ colorimetrically detecting H_2_O_2_ and ascorbic acid (AA) and electrochemically detecting dopamine. Reproduced with permission from ref. [126]. Copyright (2014) Royal Society of Chemistry; (**B**) Schematic illustration of the colorimetric biosensor for cholesterol based on the MWCNT@rGONR heterostructures. Reproduced with permission from ref. [127]. Copyright (2015) Royal Society of Chemistry.

In an elegant work, Huang and co-workers [126] designed a multifunctional composite array, namely, three dimensional graphene network@WO_3_ nanowires (3DGN@WO_3_ NWs), for efficient peroxidase mimic. Specifically, with the CVD growth of 3DGN and hydrothermal growth of WO_3_ NWs, an interconnected network of composite array exhibited a tinted yellow appearance having pores from 100 to 300 μm in diameter (Figure 11). Interestingly, the hollow graphene bridge did not show any cracks or breaks and was *ca.* 50 μm wide where WO_3_ NWs (20 nm) were uniformly distributed. In PBS system, the 3DGN@WO_3_ NWs composite exhibited a higher catalytic activity than those of bare 3DGN and WO_3_ NWs, producing a blue color of oxidized TMB in the presence of H_2_O_2_. Based on its intrinsic peroxidase-like activity, the composite was successfully used to develop a dual recognition platform [126] for detection of H_2_O_2_ and AA (colorimetric), and dopamine (DA) (electrochemical), a neurotransmitter (Figure 12A). The colorimetric detection specificity of nanocomposite towards AA was examined in human blood serum. In particular, the composite exhibited a remarkable selectivity for AA while other substances such as fructose, choline chloride (CC), glucose, uric acid (UA), DA, Cys, and NaCl were found silent. On the other hand, a good specificity of composite to DA was realized electrochemically over other tested substances including CC, UA, Cys, AA, and NaCl. In this milieu, it should be noted that, whereas the WO_3_ NWs, as the main catalyst in both colorimetric and electrochemical sensing, maximize the reactive surface area, the 3DGN works both as an electrolyte reservoir and current collector in electrochemical detection, shortening the diffusion distance and boosting the electrical sensitivity, respectively.

Wang and co-workers [127] synthesized MWCNT@rGO nanoribbon (<30 nm wide with a few-layer) core-shell heterostructures by facile unzipping oxidation of MWCNTs (<10 nm), followed by the chemical reduction with hydrazine. The as-obtained MWCNT@rGONR heterostructures exhibited *ca.* 15.9 and 8.4 times higher peroxidase-like activity than that of bare MWCNTs and their unreduced form, respectively. Similar to the natural peroxidase, the catalytic activity of MWCNT@rGONR was found to be dependent on pH and temperature. However, the catalytic activity was more stable at high concentration of H_2_O_2_ than that of HRP. Their steady-state kinetic measurements showed that the heterostructures had higher affinity to both TMB (*K*_m_ 0.12 mM) and H_2_O_2_ (*K*_m_ 1.68 mM) relative to HRP (TMB: *K*_m_ 0.43 mM, H_2_O_2_*K*_m_ 3.7 mM), which was attributed to their larger surface area, higher conductivity, and stronger adsorption ability of TMB substrate on their surface. Furthermore, the molecular level mechanism for the peroxidase-like activity of the MWCNT@rGONR was attributed to the acceleration of their electron-transfer process and the consequent facilitation of •OH radical generation.

Based on its intrinsic peroxidase-like activity, the MWCNT@rGONR heterostructure served as an excellent platform to develop a sensitive and selective colorimetric biosensor for free cholesterol determination over normal concentrations of Cys, lactic acid, AA, and glucose interferents (Figure 12B). Table 7 summarizes the notable features of other graphene-based peroxidase-mimicking NMs as discussed above.

**Table 7:** Peroxidase-like activity of graphene-based other NMs. ^a^

Nanomaterial	Method	Substrate	LOD	Applications	Ref.
Co_3_O_4_-rGO composite	Colorimetric	TMB	0.5 μM	H_2_O_2_ detection	[121]
			1.0 μM	Glucose detection	
FA/Porous Pt NPs-GO composite	Colorimetric	TMB	125 CC	CC detection	[122]
Pt NPs-GO composite	Colorimetric	TMB, OPD, DAB, HQ, and 4-AAP phenol	1.2 nM	Cys detection	[123]
PtPdNDs-GNs composite	Colorimetric	TMB	0.1 μM	H_2_O_2_ detection	[124]
Pt-on-Pd-rGO composite	Colorimetric	TMB	0.3 μM	H_2_O_2_ detection	[125]
3DGN@WO_3_ NWs array	Colorimetric	TMB	–	H_2_O_2_ detection	[126]
			–	AA detection	
	Electrochemical		238 nM	DPA detection	
MWCNT@rGONR heterostructures	Colorimetric	TMB	10 μM	Cholesterol detection	[127]

*^a^* FA, Folic acid; CC, Cancer cells; Cys, Cysteine; NDs, Nanodendrites; GNs, Graphene nanosheets; 3DGN, three dimensional graphene network; NWs, Nanowires; DPA, Dopamine; MWCNT@rGONR, Multi-walled carbon nanotubes@reduced graphene oxide nanoribbon.

### 3.6. Graphene Quantum Dots/Graphene Dots as Peroxidase Mimetic Catalysts

Graphene quantum dots (GQDs)/graphene dots (GDs) or nano-sized graphene are zero dimensional edge-bound graphene pieces, which are usually smaller than 100 nm in size. Due to their remarkable quantum confinement and edge effect, GQDs are somewhat superior to semi-conductive quantum dots and organic dyes in terms of high photostability and low toxicity. Consequently, the last decade has witnessed their potential in the fields of biochemical sensing, bioimaging, biomedicine, and photo/electrocatalysis [128,129].

Intrigued by the intrinsic peroxidase-like activity of GO [37,98], Guo and Zhang’s group [130] prepared GQDs by mixing H_2_O_2_ and GO’s suspension followed by irradiation with a mercury lamp (365 nm, 1000 W). The as-prepared GQDs (~30 nm in average size) exhibited an excellent peroxidase-like activity producing a blue color of oxidized TMB in the presence of H_2_O_2_. Whereas the *K*_m_ values for GQDs and GO were comparable, the ν_max_ for GQDs was *ca*. four-fold than that of GO, demonstrating their higher peroxidase-like activity than that of large-sized GO nanosheets. Interestingly, GQDs retained their peroxidase-like activities even after assembling on the Au electrode and as-assembled GQDs/Au electrode exhibited great stability and performance in H_2_O_2_ detection.

Soon after this report, Li and Yang’s group [131] synthesized GQDs by hydrothermal treatment of carbon black (Vulcan XC-72) with nitric acid. The intrinsic peroxidase-like activity of as-prepared GQDs was used in the detection of H_2_O_2_, glucose, and glutathione with LOD of 10 nM, 0.5 μM, and 0.5 μM, respectively. The relatively high catalytic activity of GQDs than that of GO was attributed to their high diffusion rate and ability to combine biomolecules effectively.

In continuation of their prior work on GQDs, Guo and Zhang’s group integrated GODs with catalytically active Fe_3_O_4_ NPs via a one-step co-precipitation approach [132]. Due to the synergistic effect between GQDs and Fe_3_O_4_ (facile electron transfer from electron-rich GQDs to Fe_3_O_4_), the as-prepared GQDs-Fe_3_O_4_ 1-1 nanocomposite (1-1 represent the weight ratio of GQDs and Fe_3_O_4_) exhibited 9-, 22-, and 25-fold higher peroxidase-like activities than those of GO-Fe_3_O_4_ 1-1 composites, bare Fe_3_O_4_ NPs, and bare GQDs, respectively. Their *K*_m_ values from the Lineweaver–Burk plots showed that the GQDs-Fe_3_O_4_ composite had higher affinity to both TMB (0.05 mM) and H_2_O_2_ (0.46 mM) compared with HRP (TMB: 0.58 mM, H_2_O_2_: 1.13 mM). However, the value of *K*_cat_ for GQDs-Fe_3_O_4_ was found lower than that for HRP under virtually the same conditions. Despite this, the composite showed a remarkable stability over HRP at high temperature (20–80 °C) and could be reused after several runs, retaining *ca.* 60% of its initial activity. Based on these features, GQDs-Fe_3_O_4_ composite exhibited better or comparable catalytic efficiencies for the removal of phenolic compounds, compared to HRP. In this context, as high as >80% phenol removal was recognized using GQDs-Fe_3_O_4_ composite as catalyst.

Recently, nitrogen-doped (N-doped) GQDs have been shown to have intrinsic peroxidase-like activities and are successfully applied in the detection of glucose and H_2_O_2_[133]. Table 8 summarizes the notable features of GQDs-based peroxidase mimetics for biosensing applications.

**Table 8:** Peroxidase-like activity of GQDs/N-GQDs ^a^.

GQDs	Precursor/Synthesis method	DM	SUB	LOD	Applications	Ref.
GQDs/GQDs-Au electrode	GO/UV-irradiation	ECHEM	TMB	0.7 μM	H_2_O_2_ detection	[130]
GDs	Carbon black/Hydrothermal, 130 °C	COLM	TMB	10 nM	H_2_O_2_ detection	[131]
				0.5 μM	Glucose detection	
				0.5 μM	GSH detection	
GQDs-Fe_3_O_4_ NPs composite	GQDs + FeCl_3_ + FeSO_4_/Co-precipitation	–	TMB	–	Removal of phenolic compounds	[132]
N-GQDs	DPA, 3D NGA/Conc. H_2_SO_4_ + HNO_3_ treatment	COLM	TMB	5.3 μM	H_2_O_2_ detection	[133]
				16 μM	Glucose detection	
GQDs-ZnFe_2_O_4_ composite	ZnFe_2_O_4_, GO/UV-irradiation	ECHEM	TMB	62 aM	DNA detection	[134]
GQDs	Graphite flakes/Conc. H_2_SO_4_ + HNO_3_ treatment	COLM	TMB	6 μM	Cholesterol detection	[135]

^a^ GQDs, Graphene quantum dots; N-GQDs, N-doped graphene quantum dots; DM, Detection method; SUB, Substrate; ECHEM, Electrochemical; COLM, Colorimetric; GDs, Graphene dots as per ref. [131]; GSH, Glutathione; DPA, Dopamine; aM, Attomolar (10^−18^ M); 3D NGA, Three dimensional N-doped graphene aerogel.

## 4. Tunable Factors in the Peroxidase-Like Activities of Graphene-Based Nanomaterials (G-NMs)

The peroxidase-like activity of G-NMs can be effectively tuned depending upon several factors such as environmental conditions (pH and temperature), size-dependent properties of NMs, fabrication of binary or ternary composites (and controlling the ratio of their individual components), and smart use of modulators. For instance, owing to their smaller size, GQDs provide a high surface-to-volume ratio, resulting in their higher peroxidase-like activity than that of GO having larger size [130,131]. Likewise, the composition and morphology of G-NMs have been found critical in enhancing their catalytic activities. Specifically, among variety of examined nanocomposites in TMB oxidation reaction including platinum nanoflowers-graphene nanosheets (PtNFs-GNs), core-shell Pt@PdNFs-GNs, PtNPs-GNs, PtPd nanoalloys-GNs (PtPdNAs-GNs), and PtPd nanodendrites-GNs (PtPdNDs-GNs), the latter was found to have highest affinity to both TMB (*K*_m_ 0.04) and H_2_O_2_ (*K*_m_ 3.45). The superior catalytic activity of PtPdNDs-GNs over others or even native HRP (TMB: *K*_m_ 0.43, H_2_O_2_: *K*_m_ 3.70) can be attributed to its bimetallic composition as well as unique ND’s structure [124]. In another report, the catalytic activities of morphologically diverse Fe_3_O_4_ integrated rGO were found to be in the following order Fe_3_O_4_ NSs-rGO > Fe_3_O_4_ MSs-rGO > Fe_3_O_4_ NPHs-rGO [109]. Lee and Park’s work [114] sheds light on the positive effect of manipulating the wt % ratio of different metal NPs on GO surface, leading to the higher catalytic activities. For example, in their work, GO_MNP-10 (*K*_m_ 0.144) and GO_MNP-10-Pt-10 (*K*_m_ 0.442) have been found to have greater affinity towards TMB than those of GO_MNP-30 (*K*_m_ 0.237) and GO_MNP-30-Pt-10 (*K*_m_ 0.519).

Aside from shape, size, and composition-dependent properties, the use of certain modulators allows the peroxidase reactions to be feasible over a broad pH range, which is almost impossible with natural enzymes like HRP. In this context, a GO-Au nanocluster hybrid has been evaluated as an efficient enzyme mimic that can work even at neutral pH [100] due to the modulating effect of GO (GO can bind to the substrate and catalyst in the same nanoscale region having high surface-to-volume ratio) on the catalytic activity of GO-Au nanocluster. The mechanism of this event is similar to that of natural enzymes, which bring substrates into proximity with their active sites, leading to high catalytic activity.

Besides GO, the deposition of metal ions on the surface of nanohybrid systems can also stimulate their enzymatic activity as demonstrated by Huang and co-workers [101]. It was realized that the peroxidase-like activity of AuNPs-GO hybrids significantly decreased after their binding with antibodies. However, upon the addition of Hg^2+^, the catalytic activity of AuNPs-GO hybrids was significantly enhanced attributable to the high-specificity metallophilic Hg^2+^-Au interactions [101].

## 5. Conclusions and Future Perspectives

As in many other technical fields, the research on G-NMs as peroxidase-mimetic catalysts has seen dramatic advancement in recent years and is significantly influencing the current biotechnology and bioanalytical chemistry. Among G-NMs with diversified compositions, the binary or ternary conjugates/composites are of particular interest and have been widely studied via integration of nanoscale structures of Au, Fe_3_O_4_, Fe_2_O_3_, ZnFe_2_O_4_, Co_3_O_4_, Pt, Pd, WO_3_, and CNTs, onto the surface of GO, rGO, graphene or GQDs. Besides several distinct advantages shared with bare metal/metal oxide NMs or other carbon-based NMs such as fullerenes and CNTs, G-NMs as peroxidase mimetic catalysts are unique and highly promising due to their exceptionally large surface area as well as ease of further functionalization (Table 9).

**Table 9:** Comparison between G-NMs, CNTs and fullerenes as peroxidase mimetic catalysts ^a^.

CNMs	Key Advantages/Favorable Features	Key Disadvantages or Challenges	
G-NMs	Large surface area and abundant functional groups for further modifications, for instance, bioconjugation and as support for metal/metal oxide nanoscale structures	The frequent use of acids and other toxic chemicals in synthesis and/or functionalization is of high environmental concerns	
	Size-(shape-, structure-, composition) dependent tunable properties	Available studies suggest certain toxicity. Data not concise at the present state	
	Easy in rational design, mass production, purification, recovery and recycling	Relatively low efficiency, specificity, and selectivity than natural peroxidases as reported in several cases	
	Tunable dispersion ability in aqueous media	Limited examples for use of peroxidase substrates other than TMB	
	High operational stability and Robustness to harsh environment	Much efforts are needed to be used for diversified biosensing other than glucose and H_2_O_2_	
	Relatively low cost than natural peroxidases		
CNTs	Large surface area (relatively low than that of graphene domain)	CNTs reveal toxic effects. Cytotoxicity and a relatively high inflammatory potential is reported in several studies.	
	Size-(shape-, structure-, composition) dependent tunable properties in CNTs	Relatively high cost	
	MWCNTs are easy in rational design, mass production, purification, recovery, and recycling	Difficulties in mass production and purification of SWCNTs	
		Very limited examples and much efforts are needed to be used as peroxidase mimetic catalysts	
Fullerenes	Excellent electron acceptor	Poor water dispersibility	
	Unique chemical reactivity towards radicals	Relatively high cost	
		Difficulties in mass production	
		Limited functionalization	
		Barely used as peroxidase mimetic catalysts	

^a^ CNMs, Carbon-based nanomaterials; G-NMs, Graphene-based nanomaterials; CNTs, Carbon nanotubes; MWCNTs, Multi-walled carbon nanotubes; SWCNTs, Single-walled carbon nanotubes; TMB, 3,3′,5,5′-Tetramethylbenzidine.

Despite this, there are still some issues or much room that can be taken into account for future research so that the potential of G-NMs could be fully realized in industrial applications. In this regard, we hypothesize the following suggestions:

(1) Though graphene-based NMs are increasingly used in cellular applications, the available experimental results indicate that they are not devoid of possible risks to human health or the environment [136,137]. The surface physicochemical properties, leading to possible adverse effects, warrant further studies. In line with this, more efforts are needed to translate the prominent scientific results for practical applications.
(2) A further related issue is to avoid the use of toxic chemicals, for instance, hydrazine [127]. It is an undeniable fact that hydrazine is one of the most efficient reducing agent leading to the high C/O ratio in the reduction of GO to rGO. However, hydrazine is also highly toxic and can be readily absorbed orally, by inhalation, or even dermal routes of exposure. In addition, it potentially leads to serious environmental contamination. In this milieu, it would be worthwhile to mention here that U.S. Environmental Protection Agency (EPA) has identified hydrazine as a probable human carcinogen with a low threshold limit value (TLV) of 10 ppb.
(3) From economic viewpoint, the use of precious metals like Pt and Pd may be replaced by smart designing of novel metal alloys containing G-NMs with similar or even higher catalytic efficiencies than those of expensive metals. Besides, visible light-driven peroxidase-like activity of G-NMs [138] is another area, which should be matured in near future.
(4) Further development is needed to examine the peroxidase-mimetic activities of G-NMs in the detection of biologically important anions such as cyanide anion [139], which is known for its acute toxicity to living organisms. The other related issues are exploring efficient modulators for enhancing catalytic activities of G-NMs even at high temperature or physiological pH. Though some of the research groups have started to address this problem [100] but the research progress in this direction is still in its infancy and need more attention and efforts. Considering the Hg^2+^-stimulated catalytic activity of Ab-AuNPs-GO conjugates [101], an obvious question originates, how effective would be the anions in this regard? The other question of further interest is—would the catalytic efficiencies of G-NMs be the same with other substrates, aside from that of TMB, which is most frequently used?
(5) Alongside, the technical loopholes/misconceptions regarding the nomenclature of G-NMs is the area of considerable concern [44] and should be tackled effectively in order to maintain scientific integrity for young and future generations.

In brief, the structural features and exceptional possibility for on-demand sophisticated surface engineering of graphene and its derivatives indicate that graphene-based peroxidase mimic will continue to thrive in near future.
